# Anti-D monoclonal antibodies from 23 human and rodent cell lines display diverse IgG Fc-glycosylation profiles that determine their clinical efficacy

**DOI:** 10.1038/s41598-019-57393-9

**Published:** 2020-01-30

**Authors:** Belinda M. Kumpel, Radka Saldova, Carolien A. M. Koeleman, Jodie L. Abrahams, Agnes Hipgrave Ederveen, Kathryn L. Armour, Natalia I. Olovnikova, Gestur Vidarsson, Rick Kapur, Pauline M. Rudd, Manfred Wuhrer

**Affiliations:** 1 Bristol Institute for Transfusion Sciences, International Blood Group Reference Laboratory, NHS Blood and Transplant, Bristol, UK; 2 NIBRT Glycoscience Group, National Institute for Bioprocessing Research and Training, Dublin, Ireland; 3 Center for Proteomics and Metabolomics, Leiden University Medical Center, Leiden, The Netherlands; 4 Department of Pathology, University of Cambridge, Cambridge, UK; 5 National Research Center for Hematology, Moscow, Russia; 6 Sanquin Research and Landsteiner Laboratory, Department for Experimental Immunohematology, Academic Medical Centre, University of Amsterdam, Amsterdam, The Netherlands

**Keywords:** Glycobiology, Proteomics, Immunosuppression, Haematological diseases, Drug development

## Abstract

Anti-D immunoglobulin (Anti-D Ig) prophylaxis prevents haemolytic disease of the fetus and newborn. Monoclonal IgG anti-Ds (mAb-Ds) would enable unlimited supplies but have differed in efficacy in FcγRIIIa-mediated ADCC assays and clinical trials. Structural variations of the oligosaccharide chains of mAb-Ds are hypothesised to be responsible. Quantitative data on 12 Fc-glycosylation features of 23 mAb-Ds (12 clones, 5 produced from multiple cell lines) and one blood donor-derived anti-D Ig were obtained by HPLC and mass spectrometry using 3 methods. Glycosylation of mAb-Ds from human B-lymphoblastoid cell lines (B) was similar to anti-D Ig although fucosylation varied, affecting ADCC activity. *In vivo*, two B mAb-Ds with 77–81% fucosylation cleared red cells and prevented D-immunisation but less effectively than anti-D Ig. High fucosylation (>89%) of mouse-human heterohybridoma (HH) and Chinese hamster ovary (CHO) mAb-Ds blocked ADCC and clearance. Rat YB2/0 mAb-Ds with <50% fucosylation mediated more efficient ADCC and clearance than anti-D Ig. Galactosylation of B mAb-Ds was 57–83% but 15–58% for rodent mAb-Ds. HH mAb-Ds had non-human sugars. These data reveal high galactosylation like anti-D Ig (>60%) together with lower fucosylation (<60%) as safe features of mAb-Ds for mediating rapid red cell clearance at low doses, to enable effective, inexpensive prophylaxis.

## Introduction

Anti-D immunoglobulin (anti-D Ig, RhIG) is a very safe and effective prophylactic therapy to prevent haemolytic disease of the fetus and newborn (HDFN). After its introduction 50 years ago, deaths are now rare, approximately 0.02 per thousand births, a reduction of about 98% since 1950 when mortality from HDFN was about 10% of perinatal deaths^1^.

However, in low- or middle-income countries HDFN still affects thousands of babies annually^2^. Worldwide estimates for 2010 were 141,000 fetal and neonatal deaths and 27,000 cases of kernicterus caused by bilirubin toxicity leading to a high risk of lifelong neurological dysfunction^3^. Many countries have insufficient, sporadic or no anti-D prophylaxis due to its unavailability, high cost^4,5^ or insufficient public healthcare organisation or resources^2^.

Anti-D Ig preparations consist of IgG fractionated from pooled plasma of hyperimmunised D-negative donors. These IgG preparations have multiple anti-D specificities and affinities. Relatively low doses of this poly-clonal anti-D (100^6^–300^1^ µg anti-D) are administered antenatally and/or postnatally to susceptible women, which are D-negative with a D-positive fetus or baby. Fetal blood may leak into maternal blood by fetomaternal haemorrhage (FMH) through the placenta, occasionally during pregnancy but more often after parturition^7,8^ with fetal bleeds then usually of greater volume but rarely exceeding 5 mL. FMH is the cause of maternal alloimmunisation^9^. Unique immunologic changes in pregnant and postpartum women induced by placental syncytiotrophoblast microparticles^10,11^ and pregnancy hormones^12^ ensure they make strong protective antibody responses to foreign antigens which include, unfortunately, responses to allogeneic blood cells^13^. Prophylactic anti-D accelerates the clearance of fetal D-positive red blood cells (RBC) from the maternal circulation^14^, preventing D-immunisation which may otherwise result in HDFN. Fetal RBC with bound anti-D are removed by the spleen^15^ via macrophage IgG Fc receptor (FcγR)IIIa recognition of cell-bound anti-D^16,17^ which triggers phago-cytosis and non-inflammatory intracellular destruction^18^. Consequently, FcγRIIIa-mediated antibody-dependent cellular-cytotoxicity (ADCC) assays are a good predictor of red cell clearance by IgG anti-D^19^.

**Figure 1:**
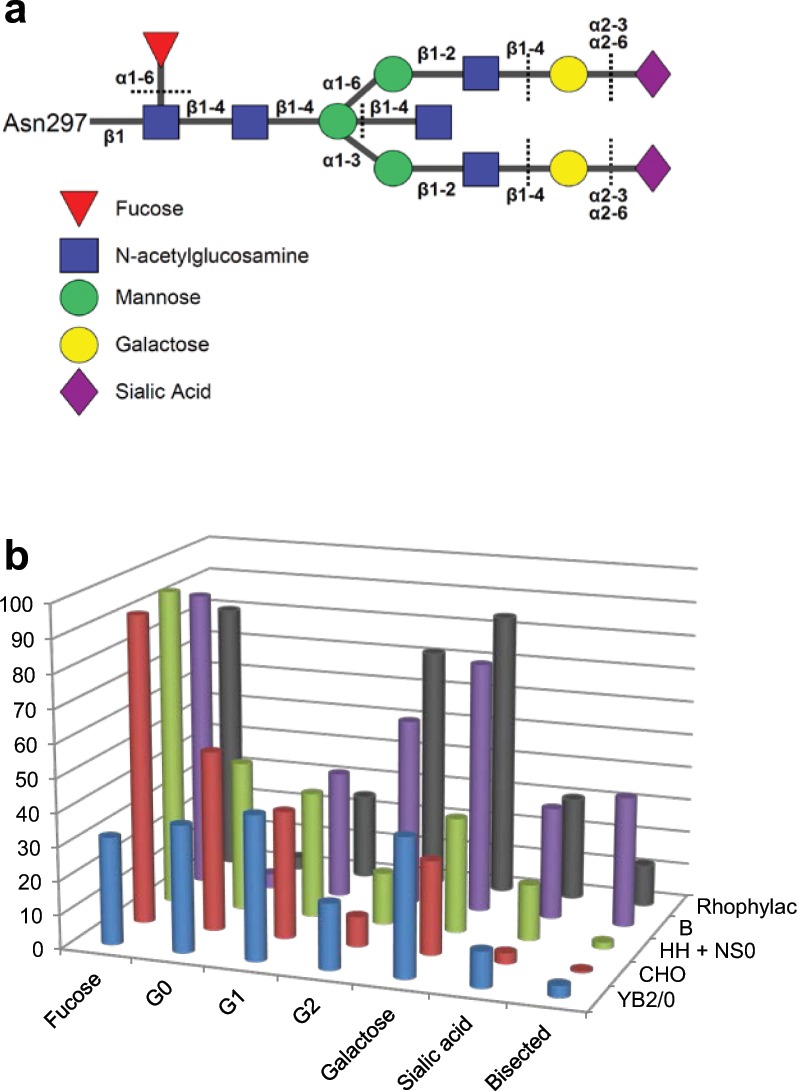
Glycosylation of anti-D IgG-Fc. (**a)** Cartoon of the branched oligosaccharide chain covalently attached to Asn297 of each Fc in the Cγ2 domain of IgG. The sugar linkages are shown. Dotted lines indicate the structures that may or may not be present on different glycans. **(b)** Summary of glycosylation of anti-Ds. Bar chart showing the average glycosylation of IgG1 mAb-Ds produced from human B, mouse HH + NS0, hamster CHO and rat YB2/0 cell lines, compared to Rhophylac anti-D. The percentage of glycans with fucose, galactose, agalactose (G0), monogalactose (G1), digalactose (G2), sialic acid and bisecting GlcNAc of the total samples of each cell line group is illustrated.

Anti-D monoclonal antibodies (mAb-Ds) would be safe, inexpensive, standardised products potentially capable of replacing anti-D Ig. Several groups have made mAb-Ds and tested them in relevant biological assays *in vitro* and in human studies of RBC clearance and prevention of D-immunisation. Surprisingly, mAb-Ds have shown great variability in these studies but none have yet had equivalent activity to anti-D Ig^19^. It was hypothesised that this may be due to differences in their glycosylation^19,20^, i.e. the composition and linkage of sugars in the oligosaccharide chains attached to the Fc portion of IgG^21^. Human IgG has a highly conserved branched glycan chain covalently attached to Asn297 of each Cγ2 domain (Fig. 1a). This glycan contains variable amounts of fucose, galactose, sialic acid, and bisecting N-acetylglucosamine (GlcNAc). Remarkably, we have found that alloimmune IgG1 responses against platelets and RBC antigens, including anti-D, are characterised by low fucosylation and increased galactosylation in most sera^22–24^ as well as in the anti-D component of anti-D Ig preparations^25^.

For this study, extensive glycosylation analyses of an anti-D Ig preparation and 23 mAb-Ds produced from cell lines of four species (human, mouse, hamster and rat) were performed independently by two research groups. The mAb-Ds comprised 12 unique clones; 5 of these clones produced anti-D from 2–4 cell lines. Fourteen of these mAb-Ds had been previously tested in 10 clinical studies. Retrospective data analysis of clearance of D-positive RBC by all 14 mAb-Ds and of prevention of D-immunisation by 6 of them is presented here. Glycosylation of IgG1 and IgG3 anti-Ds was determined by high-performance liquid chromatography (HPLC) analysis of fluorescently labelled N-glycans, before and after exoglycosidase digestion. In a second approach, N-glycans from total IgG1 anti-D were analysed by mass spectrometry (MS) after ethyl esterification of sialic acids. In a third approach, MS was also used to analyse IgG1 anti-D Fc-glycopeptides. ADCC assays were performed and glycan structural data were linked to ADCC activity. The data imply that cell line-dependent variations in glycosylation between mAb-Ds had a major influence on biological activity.

## Results

### Glycosylation varied markedly among the anti-Ds

Results from the three analytical methods determining 12 glycosylation features were similar (Tables 1–3) and concurred with earlier data from small-scale studies (Supplementary Table S1). Glycosylation of intravenous immunoglobulin (IVIG) was similar to other products^26^ and that of the IgG anti-D purified from Rhophylac 300 anti-D Ig (Rhophylac) agreed with an earlier report^25^. Glycosylation profiles of the mAb-Ds depended on the producer cell lines. The data is summarised in Fig. 1b.

**Table 1:** Analysis of total fucosylation, bisecting N-acetylglucosamine and alpha-galactose of Fc-glycans from IVIG, Rhophylac anti-D and mAb-Ds.

Antibody	Cell line	IgG sub- class	% Lysis in ADCC at 2500 ng/mL	% Fucosylation			% Bisecting GlcNAc			% α-Galactose	
				Flu	Eth	GP	Flu	Eth	GP	Flu	Eth
IVIG Rhophylac		1>2>3>4 1	88.2	86.8 79.8	95.7 82.1	91.6 78.0	15.6 11.0	18.4 12.0	14.2 13.2	0.0 0.0	0.0 0.0
BRAD3lab BRAD3clin mBRAD3 BRAD5lab BRAD5clin mBRAD5 G7 G12 G108 AB5 JAC10	B B B B B B B B B B B	3 3 3 1 1 1 1 1 1 1 1	6.5 25.7 95.9 55.1 10.9 8.8	87.7 77.0 78.9 67.8 81.2 87.3 92.5 91.6	68.9 94.0 98.9 99.5 90.2 96.6 98.5	63.9 87.2 94.9 93.9 86.8 96.3 95.3	65.6 62.0 63.9 48.8 65.8 35.5 13.3 27.0	53.9 46.8 12.8 34.2 23.2 58.7 25.2	53.9 42.9 15.0 34.8 22.9 54.6 23.6	0.0 0.0 0.0 0.0 0.0 0.0 0.0 0.0	1.1 2.3 0.0 0.0 0.5 1.5 2.3
Fog1 Fog1 G7 G12 AD1	NS0 HH HH HH HH	1 1 1 1 1	5.4 8.4 47.8 28.5	100.0 83.6 86.0 96.8	99.2 100.0 92.4 93.0 97.9	92.3 95.9 90.2 90.5 96.6	2.3 0.0 2.5 2.6	1.1 0.0 2.4 2.3 0.4	4.5 2.5 3.6 3.6 1.5	4.4 1.0 3.5 0.6	5.3 0.0 3.5 0.4 3.2
MonoRho rBRAD3 rBRAD5	CHO CHO CHO	1 3 1	28.0 17.3 32.0	92.3 73.6 90.9		92.7	0.0 0.0 0.0		2.5	0.0 0.0 0.0	
Fog1 G12 R297 Fog1Δnab	YB2/0 YB2/0 YB2/0 YB2/0	1 1 1 1 null	107.3 111.1	17.5 20.3	24.2 23.0 31.6	27.2 32.9 35.4 45.6	0.0 7.7	1.0 9.4 0.0	2.7 12.2 4.5 1.5	0.0 0.0	0.0 0.0 0.0

Glycosylation analysis methods used were Flu (NP-HPLC analysis of fluorescently labelled, released N-glycans), Eth (analysis of released N-glycans by MALDI-TOF-MS after ethyl esterification) and GP (analysis of IgG Fc-glycopeptides by LC-MS).

**Table 2:** Analysis of total galactosylation and agalactosyl (G0), monogalactosyl (G1) and digalactosyl (G2) of Fc-glycans from IVIG, Rhophylac anti-D and mAb-Ds.

Antibody	Cell line	IgG sub- class	% Galactosylation			% G0			% G1			% G2		
			Flu	Eth	GP	Flu	Eth	GP	Flu	Eth	GP	Flu	Eth	GP
IVIG Rhophylac		1>2>3>4 1	58.8 82.3	62.1 85.6	61.1 84.4	21.2 4.6	17.3 1.7	18.4 3.3	46.9 26.2	41.1 24.2	40.8 24.4	35.4 69.2	41.6 73.4	40.7 72.2
BRAD3lab BRAD3clin mBRAD3 BRAD5lab BRAD5clin mBRAD5 G7 G12 G108 AB5 JAC10	B B B B B B B B B B B	3 3 3 1 1 1 1 1 1 1 1	68.6 60.7 56.8 78.5 64.0 53.5 82.6 84.2	73.7 59.9 82.7 83.4 73.3 70.8 83.8	77.8 66.4 82.6 83.8 74.5 72.5 84.9	11.4 16.2 17.8 0.0 12.1 14.3 2.2 1.1	2.7 5.0 1.5 0.8 3.9 3.5 0.0	0.0 6.4 2.8 1.3 5.5 5.2 3.0	35.0 38.8 44.8 42.9 41.7 45.7 30.3 29.5	41.9 55.5 30.5 31.7 41.4 46.8 23.8	39.4 45.4 28.7 29.4 39.7 44.3 23.8	51.0 41.3 34.4 57.1 43.1 30.6 67.5 69.4	52.8 32.1 67.4 67.5 52.6 47.4 71.8	58.1 43.8 68.3 69.1 54.7 50.3 73.0
Fog1 Fog1 G7 G12 AD1	NS0 HH HH HH HH	1 1 1 1 1	40.3 27.3 41.2 20.2	42.9 20.9 39.8 22.0 38.5	45.4 24.3 44.7 28.5 37.3	38.8 59.8 37.2 65.5	27.3 63.3 29.7 52.1 32.6	34.1 59.3 33.1 55.3 40.7	41.8 26.0 43.1 28.7	46.2 31.6 45.1 29.4 37.6	39.8 31.9 43.9 30.4 43.1	19.4 14.3 19.7 5.9	19.8 5.2 17.3 7.3 19.7	25.5 8.4 22.8 13.3 15.8
MonoRho rBRAD3 rBRAD5	CHO CHO CHO	1 3 1	26.5 32.6 25.9		32.7	54.4 49.0 55.6		46.7	38.3 32.7 37.0		38.8	7.3 16.2 7.4		13.3
Fog1 G12 R297 Fog1Δnab	YB2/0 YB2/0 YB2/0 YB2/0	1 1 1 1 null	10.1 54.2	16.2 56.7 44.3	19.7 61.5 45.9 45.7	81.0 20.2	71.5 16.2 27.6	68.5 17.0 29.7 30.6	17.8 51.2	24.7 54.9 56.0	23.2 42.4 48.5 47.4	1.2 28.6	3.8 29.3 16.3	8.1 40.3 21.6 22.0

Glycosylation analysis methods used were Flu (NP-HPLC analysis of fluorescently labelled, released N-glycans), Eth (analysis of released N-glycans by MALDI-TOF-MS after ethyl esterification) and GP (analysis of IgG Fc-glycopeptides by LC-MS).

**Table 3:** Analysis of total sialylation, sialic acid types and linkages of Fc-glycans from IVIG, Rhophylac anti-D and mAb-Ds.

Antibody	Cell line	IgG sub- class	% Sialylation			% N-Acetyl	% N-Glycolyl	Linkage		% α2,3		% α2,6	
			Flu	Eth	GP	Eth	Eth	Flu	Eth	Flu	Eth	Flu	Eth
IVIG Rhophylac		1>2>3>4 1	19.0 27.2	23.3 30.7	19.2 32.9	23.3 30.7	0.0 0.0	α2-6 α2-6	α2-3,6	0.0 0.0	0.0 0.7	19.0 27.2	23.3 30.0
BRAD3lab BRAD3clin mBRAD3 BRAD5lab BRAD5clin mBRAD5 G7 G12 G108 AB5 JAC10	B B B B B B B B B B B	3 3 3 1 1 1 1 1 1 1 1	28.6 32.4 27.5 23.5 33.7 18.7 35.3 34.6	27.9 24.2 35.9 35.1 32.8 27.9 45.6	32.0 29.3 38.9 39.8 34.9 30.5 45.5	27.9 24.2 35.9 35.1 32.8 27.9 45.6	0.0 0.0 0.0 0.0 0.0 0.0 0.0	α2-3,6 α2-3,6 α2-3,6 α2-3,6 α2-3,6 α2-3,6 α2-3,6 α2-3,6	α2-3,6 α2-3,6 α2-3,6 α2-6 α2-3,6 α2-3,6 α2-6	12.0 10.1 14.2 4.2 10.3 10.2	0.7 0.6 0.6 0.0 2.3 1.4 0.0	16.6 17.4 19.5 14.5 25.1 24.4	27.2 23.6 35.3 35.1 30.5 26.5 45.6
Fog1 Fog1 G7 G12 AD1	NS0 HH HH HH HH	1 1 1 1 1	5.8 11.7 22.8 10.2	7.5 6.6 29.1 15.8 30.2	9.8 10.7 25.7 18.5 19.3	0.0 0.0 0.0 0.0 0.0	7.5 6.6 29.1 15.8 30.2	α2-3,6 α2-3,6 α2-3,6 α2-6	α2-3,6 α2-6 α2-3,6 α2-3,6 α2-3,6	0.9 2.5 2.8 0.0	1.4 0.0 1.7 1.7 1.6	4.9 9.3 20.0 10.2	6.2 6.6 27.4 13.9 28.6
MonoRho rBRAD3 rBRAD5	CHO CHO CHO	1 3 1	0.0 5.2 3.5		5.9			0.0 α2-3 α2-3		0.0 5.2 3.5		0.0 0.0 0.0	
Fog1 G12 R297 Fog1Δnab	YB2/0 YB2/0 YB2/0 YB2/0	1 1 1 1 null	0.0 14.3	1.7 17.0 6.3	4.4 22.3 9.8 14.6	1.7 10.4 6.3	0.0 6.7 0.0	0.0 α2-3,6	α2-6 α2-3,6 α2-6	0.0 3.7	0.0 1.1 0.0	0.0 10.6	1.7 15.9 6.3

Glycosylation analysis methods used were Flu (NP-HPLC analysis of fluorescently labelled, released N-glycans), Eth (analysis of released N-glycans by MALDI-TOF-MS after ethyl esterification) and GP (analysis of IgG Fc-glycopeptides by LC-MS).

N-Acetyl, N-acetylneuraminic acid (NeuAc); N-Glycolyl, N-glycolylneuraminic acid (NeuGc).

### Fucosylation

Fucosylation was high in IVIG (91%), Rhophylac (80%), and mAb-Ds from most human B lymphoblastoid cell lines (B) (67–95%), mouse NS0 and heterohybridoma (HH) cell lines (90–97%) as well as Chinese hamster ovary (CHO) cell lines (74–93%). In contrast, mAb-Ds produced from rat YB2/0 cell lines had much lower fucosylation (23–46%) (Table 1).

### Galactosylation

The number of galactose residues on the branched oligosaccharide varied greatly. Galactosylation of Rhophylac (84%), most B mAb-Ds (mean 71%) and IVIG (61%) was markedly higher than for rodent cell mAb-Ds (mean 35%). The percentages of G0 (agalactosyl IgG), G1 (monogalactosyl IgG) and G2 (digalactosyl IgG) were calculated for each method. Mean G2 values were over 3 times higher for human (72% Rhophylac, mean 52% B mAb-Ds) than for rodent cell (mean 16%) anti-Ds. G1 values for Rhophylac were slightly lower than for IVIG and most mAb-Ds. Strikingly, nearly half the Fc-glycans of rodent cell mAb-Ds had no galactose (mean 43% G0), contrasting greatly with very low levels of G0 in Rhophylac (3.2%) and IgG1 B mAb-Ds (mean 4.5%) (Table 2).

### Sialylation

Sialic acid is linked to galactose on IgG Fc oligosaccharides. Sialylation was approximately one third that of galactosylation for IVIG and Rhophylac and was relatively higher for B and HH mAb-Ds but lower for NS0, CHO and YB2/0 mAb-Ds (half had <10% sialylation) (Fig. 1b). IVIG, Rhophylac, B mAb-Ds and two YB2/0 mAb-Ds expressed only N-acetylneuraminic acid (NeuAc) while NS0 and HH cell mAb-Ds had only N-glycolylneuraminic acid (NeuGc) and G12-yb2/0 had both types of sialic acids. The linkage of sialic acid to galactose was predominantly α2,6 in IVIG, Rhophylac, B, NS0, HH and YB2/0 mAb-Ds but only α2,3 was detected in CHO mAb-Ds (Table 3).

### Bisection

Compared to Rhophylac and other prophylactic anti-Ds^25^, levels of bisecting N-acetylglucosamine (GlcNAc) were much higher for all B mAb-Ds but lower for all rodent cell mAb-Ds, in some cases undetectable. G7-b and G12-yb2/0 had values closest to Rhophylac (Table 1).

### Alpha galactose

Low levels of an additional galactose, Galα1–3Gal (α-Gal), were detected by fluorescently-labeled glycans and ethyl esterification on NS0 and HH mAb-Ds and on some human B mAb-Ds by ethyl esterification only (Table 1).

### Culture methods affected glycosylation

Galactosylation was higher (77%) and fucosylation lower (67%) in BRAD5lab-b derived from low cell density flask culture than in the same mAb-D produced from high cell density hollow fibre bioreactors, BRAD5clin-b and mBRAD5-b. These had 64% and 60% galactosylation and 81% and 90% fucosylation, respectively.

### Functional activity of mAb-Ds in ADCC assays was inversely related to fucosylation

**Figure 2:**
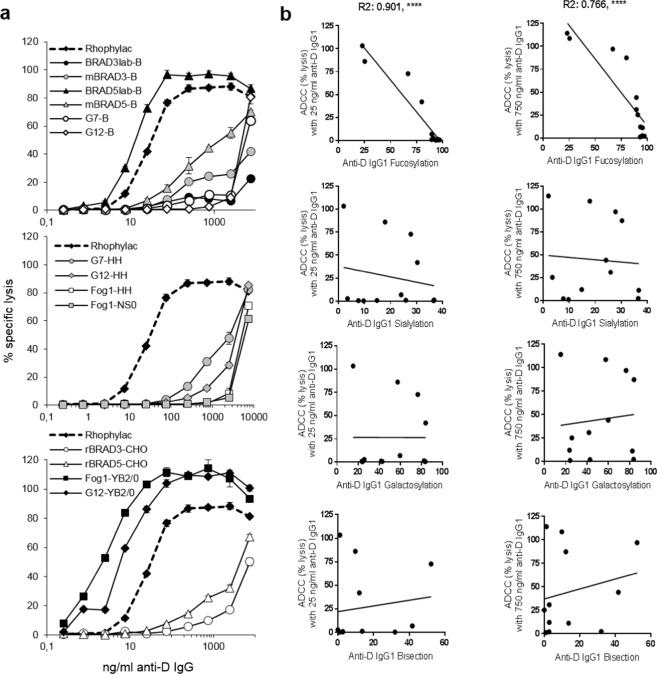
Functional activities of anti-D in ADCC assay. (**a)** The anti-D dependent lysis of red cells by NK cells is shown throughout the dilution range of antibody activity. Rhophylac anti-D is compared to mAb-Ds from human B cell lines, mouse cell lines, and hamster CHO and rat YB2/0 cell lines. Error bars indicate standard deviation (±s.d.). **(b)** Correlation between ADCC activities of anti-Ds and their glycosylation. Comparison is made of the percentage fucosylation, sialylation, galactosylation and bisecting GlcNAc with the percentage lysis of the anti-Ds (mAb-Ds and Rhophylac anti-D) shown at 25 ng/ml (left column) and 750 ng/ml (right column). The Pearson correlation coefficients for ADCC lysis and fucosylation are shown on the graphs, both were significant to p < 0.0001. There was no correlation between ADCC activity and percentage sialylation, galactosylation or bisection.

The anti-Ds varied greatly in sensitive natural killer (NK) cell FcγRIIIa-dependent ADCC activity. Four anti-Ds, namely Rhophylac, BRAD5lab-b, Fog1-yb2/0 and G12-yb2/0 exhibited sigmoidal dose-response curves and mediated high potency at relatively low concentrations (<100 ng/ml). All other mAb-Ds tested elicited less activity even at saturating concentrations. At 250 ng/ml, BRAD5lab-b had 66% and 85% greater efficacy than mBRAD5-b and rBRAD5-cho, respectively, while Fog1-yb2/0 and G12-yb2/0 were 95–99% more efficient than their B, NS0 and HH forms in ADCC assays (Fig. 2a).

Comparison of activity with fucosylation of IgG1 anti-Ds revealed a strong negative correlation with a striking reduction in activity when fucosylation was >80% (Fig. 2b, top panels). At 25 ng/ml, which is the mean maximum physiological concentration after injection of anti-D Ig^27^, only four anti-Ds (Fog1-yb2/0, G12-yb2/0, BRAD5lab-b as well as Rhophylac) were active (Fig. 2b, top left panel). At 0.75–2.5 ng/ml (approximate range of detectable anti-D 10 weeks after injection^27^) only these three mAb-Ds were active (Fig. 2a). There was no significant relationship between ADCC activity and sialylation, galactosylation or bisecting GlcNAc of the anti-Ds at either 25 ng/ml or 750 ng/ml (Fig. 2b, lower panels). However, mBRAD5-b had equal fucosylation as G7-hh (89.5%) but higher ADCC (Fig. 2a) and more galactosylation (60% versus 42%) and bisecting GlcNAc (42% versus 2.8%), thus these sugars may have led to enhanced mBRAD5-b interactions with FcγRIIIa. The IgG3 mAb-Ds, mBRAD3-b and rBRAD3-cho, had lower ADCC activity than equivalent forms of BRAD5 (Fig. 2a) despite lower fucosylation.

### Efficacy of mAb-Ds in human studies was affected by glycosylation

In autologous studies, clearance of D-positive RBC was more rapid with YB2/0 mAb-Ds (Fog1^28^ and R297^29^) than anti-D Ig, fast with BRAD3clin-b^30^, slower with BRAD3 + BRAD5 (B and CHO)^31^ and very slow with Fog1-hh^30^. Clearance by Fog1-hh^30^ and Fog1-yb2/0^28^ was markedly dissimilar despite identical amino acid sequence, which coincided with much lower fucosylation of Fog1-yb2/0. Unexpectedly, Fog1Δnab-yb2/0 (lacking FcγR interactions^32^) also cleared RBC^28^. Fucosylation of 1:3 blends of BRAD3 + BRAD5 (B and CHO) was identical (87%) but clearance was slightly greater with mBRAD3-b + mBRAD5-b^31^ having increased galactosylation, sialylation and bisection as compared to the CHO-cell produced variants of these antibodies (Table 4).

**Table 4:** Clearance of autologous D-positive RBC precoated with anti-D in D-positive subjects.

Ref-erence	Anti-D	% Fucosyl-ation	Comments on clearance	Summary of clearance	Dose-response effect	Number of subjects
30 30	BRAD3clin-b Fog1-hh	77 93	Very rapid initially then slowed Very slow, incomplete by 24 h	++++ +	Yes No	6
31 31	mBRAD3-B + mBRAD5-b rBRAD3-cho + rBRAD5-cho	85 (mean) 83 (mean)	Rapid initially then slowed Rapid initially then markedly slowed	+++ ++		6 6
28 28	Fog1-yb2/0 Fog1Δnab-yb2/0 (null)	23 46	Extremely rapid, complete by 4 h, some febrile reactions More than 50% RBC cleared by 4 h	+++++ +++	No	5 6
29 29	R297-yb2/0 Rhophylac 300	34	Extremely rapid, complete by 72 h Rapid, almost complete by 96 h	+++++ ++++	Yes Yes	6 6

Using D-negative subjects, RBC clearance was slightly less effective with BRAD3clin-b and BRAD5clin-b than anti-D Ig^33–35^, regardless of the order of injection of anti-D and RBC - anti-D first^33,34^ or RBC first^35^. However, for HH^36,37^ and CHO^38^ mAb-Ds injected after RBC, clearance was slow and variable although for two subjects given anti-D (G12-hh) before RBC^37^, clearance was rapid (Table 5).

**Table 5:** Clearance of allogeneic D-positive RBC in D-negative subjects by anti-D and subsequent protection against D-immunization (prophylaxis).

Ref-erence	Anti-D	% Fuco-sylation	Comments on clearance	Summary of clearance	Number of naïve subjects	Percentage forming anti-D before re-immunizing IgG IgM	Number of responders protected
33 33 33 34 35	BRAD3clin-b BRAD5clin-b Anti-D Ig BRAD3clin-b + BRAD5clin-b BRAD3clin-b + BRAD5clin-b	77 81 80 (mean) 80 (mean)	Mean t_50%_ = 12.7h, dose-response Mean t_50%_ = 5.9h, dose-response Mean t_50%_ = 5.0h Mean t_50%_ = 9.6h Complete by 72h in 87% (81/93) subjects	++ +++ ++++ +++ +++	10 8 8 8 93	0% 0% 0% 0% 1.7%	3 of 3 1 of 1 1 of 1 2 of 2 20 of 22
36 36	AD1-hh + AD3-hh Anti-D Ig	97	Variable, incomplete Rapid	+/++ ++++	5 4	60% 0%	
37 37 37 37 37	G7-hh G7-hh + G12-hh G12-hh G12-hh Anti-D Ig	90 91 93 93	Moderate Moderate Variable, incomplete Rapid Rapid	+++ ++/+++ +/++ ++++ ++++	6 3 10 2 2	67% 50% 100% 100% 60% 40% 0% 0% 0% 0%	
38 38	MonoRho-cho Rhophylac 300	93	Slow, very variable t_50%_ = 2-203h no dose-response Rapid	+/++/+++ ++++	31 15	0% 0%	

In the following six months, immune IgG anti-D was detected 4–24 weeks after RBC injection in 0.8% (1 of 119) subjects given B mAb-Ds^33–35^, 62% receiving HH mAb-Ds^36,37^, 0% injected with MonoRho-cho^38^ and 0%^33,36–38^ administered anti-D Ig (Table 5). Unexpectedly, IgM anti-D was also detected in 77% of the immunised subjects receiving G7-hh and G12-hh^37^. Assessing the efficacy of MonoRho-cho was hindered by “rescue prophylaxis” (anti-D Ig) given to 7 subjects with slow RBC clearance^38^. After protracted studies of BRAD3clin-b and BRAD5clin-b which included two further RBC immunisations at 6 and 9 months, 93% (26 of 28) of the responders who developed anti-D were shown to have been protected from becoming D-immunised to the initial RBC injection by these B mAb-Ds^33–35^ (Table 5).

## Discussion

Fc N-glycans extend from Asn297 in the N-terminal lower hinge regions of IgG into the Cγ2 inter-domain space, forming weak interactions with the protein^39–41^. Both the Cγ2 domains and the glycans are to some extent mobile and asymmetric^40,41^. FcγRIIIa binds to both lower hinge regions^42^. Glycan composition may affect the N-terminal conformation or the relative orientation or mobility of Cγ2 domains, modifying affinity for FcγRs^41^, although the precise mechanisms remain undefined^43^. Glycosylation of Fab (antigen-binding) regions of anti-D is unlikely because the integral membrane RhD proteins are surrounded by negatively charged glycoproteins in the RBC membrane glycocalyx, constricting access to the antigen^44^. In support of this, we recently found a strong selection against the formation of Fab-glycans during hypermutation in anti-D^45^.

Anti-D represents an ideal IgG for structure/function investigation and is unique because mAb-Ds from six types of cell lines could be compared with anti-D Ig synthesised by plasma cells for both *in vitro* functional activity and *in vivo* clinical data. The glycosylation of anti-Ds was heterogenous, defined by the producer cells, and influenced their biological and clinical activities.

The contribution of individual sugars to functional activity of IgG is becoming increasingly clear and may prove highly relevant for mAb-Ds.

Fucose (proximal to Asn297) was the first glycan variant found to affect the activity of human IgG1, inhibiting FcγRIIIa-mediated ADCC^46^ and phagocytosis^22^. It causes steric inhibition of the Fc-FcγRIIIa interaction^47^. Afucosylated IgG has high affinity for FcγRIIIa^22,47^ displacing plasma IgG and enabling ADCC at low concentrations^48^. Many alloantibodies, but not all, have considerably less fucose than total IgG1^22–25,49^. Fucosylation of anti-D in 11 prophylactic preparations was 56%-91%, while for Rhophylac this was 81%^25^.

The low fucosylation (<35%) of YB2/0 mAb-Ds enabled them to be highly active (effective ADCC and fast red cell clearance) but fucosylation was too high in most of the B, HH and CHO mAb-Ds for high affinity ADCC responses and accelerated red cell clearance. BRAD5lab-b was effective but when produced for clinical use fucosylation was elevated and this came with a lower efficacy. Surprisingly, most B mAb-Ds (including AB5 and JAC10) have minimal ADCC^20,50^ perhaps because EBV immortalises immature circulating B cells synthesising highly fucosylated IgG whereas plasma cells secreting low fucosylated protective antibodies lack EBV receptors^51^. The high fucosylation of these B mAb-Ds is likely to explain their low ADCC.

Galactosylation of IgG has been found to be regulated by estrogens^52^, increased during pregnancy^53^ and associated with pregnancy-induced remission of rheumatoid arthritis^54^. Agalactosylation levels of IgG have been reported to be two-fold higher in patients with rheumatoid arthritis than controls^55^ and associated with markers of inflammation^56,57^. Low galactosylation of anti-proteinase 3 autoantibodies correlated with inflammatory cytokines^58^. In our experiments, galactosylation correlated with moderately increased ADCC of BRAD5lab-b^59^, glycoengineered IgG1^60^ and hypo-fucosylated anti-D^61^. Thus, the wide range of galactosylation ranging from Rhophylac and IgG1 B mAb-Ds (60–84% galactosylation with <15% G0) to rodent mAb-Ds (15–57% galactosylation with 18–70% G0) may impact antibody function in various ways.

Sialic acid has been described not to alter ADCC activity of IgG1^60^. Similarly, sialylation of anti-D had little or no effect on FcγR binding^61^ or macrophage phagocytosis of sensitised RBC^62^. Low sialylation of CHO and YB2/0 mAb-Ds (mean 8%) and lack of the α2,6-linkage on CHO mAb-Ds may make them liable to inflammatory responses. In addition, non-human Neu5Gc on NS0, HH and G12-yb2/0 mAb-Ds may be immunogenic^63,64^.

The biological relevance of bisecting GlcNAc is uncertain. Increasing it has been reported to enhance FcγRIIIa-mediated ADCC, possibly by affecting fucosylation^65,66^, but recently, little effect has been found for monoclonal anti-D^61^. Bisection was very high on most B mAb-Ds.

Alpha-galactose (α-Gal epitope: Galα1–3Galβ1-(3)4GlcNAc-R) is synthesised by all mammals except humans, apes and Old World monkeys, which produce anti-Gal^67^, comprising ~1% of human IgG^68^. Humans also have high concentrations of anti-Neu5Gc (usually higher than anti-B (blood group antibody))^64^. These natural antibodies may bind mAbs expressing xenogeneic α-Gal and Neu5Gc epitopes, forming immune complexes and increasing uptake of target cells to antigen-presenting cells and immunogenicity. All NS0 and HH mAb-Ds expressed α-Gal, as reported previously^69^, and Neu5Gc, also found on mAbs from some murine myelomas^70^. These xenogeneic epitopes may have caused HH mAb-Ds to stimulate anti-D responses, not prevent them. The findings by one laboratory of low amounts of α-Gal on some human B mAb-Ds cultured in the absence of animal material are unexpected and should be taken with caution as further studies would be needed to substantiate this.

IgG3 anti-D comprises 10% of the anti-D in prophylactic preparations on average^71^ but is relatively inefficient in ADCC compared to IgG1 anti-D as is BRAD3 (IgG3) compared to BRAD5 (IgG1)^72^. ADCC assays measured FcγRIIIa-mediated haemolysis by NK cells although *in vivo* FcγRIIIa-bearing splenic macrophages phago-cytose anti-D opsonised RBC^16,17^. *In vivo*, BRAD3clin-b efficiently cleared RBC^30,33^. *In vitro*, RBC opsonised with BRAD3lab-b had greater mean binding to splenic macrophages in cryostat sections than BRAD5lab-b opsonised RBC (58.6 and 25.8 respectively)^73^. Additionally, using monocyte-derived macrophages, IgG3 mAb-Ds mediated higher ADCC than IgG1 mAb-Ds (96% versus 26%)^50^. This difference in activity may be explained because glycoforms of FcγRIIIa vary between NK cells and monocytes^74^ which may affect affinity to IgG subclasses and binding of differentially glycosylated IgG. Recognition of afucosylated IgG by FcγRIIIa is in part mediated through carbohydrate-carbohydrate interactions involving the N162-glycan found on this receptor^47^.

The role of cells and FcγRs in RBC clearance is becoming clearer. *In vitro*, phagocytosis of anti-D opsonised RBC by monocytes is mediated by FcγRI, with the extent of phagocytosis proportional to anti-D coverage on RBCs^50^. FcγRI is also present on splenic red pulp macrophages and although at low expression compared to FcγRIIIa, it gives a major contribution to phagocytosis^75^. This may be due to upregulation of surface expression of FcγRI after stimulation of FcγRIIIa by binding opsonised RBC or by inflammation^75^. Thus *in vivo*, it is likely that opsonised RBC are selected and captured by splenic macrophages through FcγRIIIa binding afucosylated anti-D followed by FcγRI-mediated internalisation. The spleen has the capacity to phagocytose all the fetal RBC in the majority of FMH (volumes over 20 ml fetal RBC are exceptional) without producing spherocytes or free haemoglobin. RBC with the highest opsonisation will be removed first, the rate of clearance correlating with the amount of RBC-bound anti-D^14^ (and indirectly to D antigen levels), resulting in progressive slowing of clearance of RBC with decreasing anti-D opsonisation. Notably, antigen masking may only occur to a minor extent, as doses of anti-D cover only about 8%–20% of D antigen sites on RBC^76^.

Although the mechanism of anti-D prophylaxis has not been fully elucidated, clinical observations and studies performed after the introduction of anti-D Ig suggest it elicits some immunomodulatory processes. (a) Prophylactic anti-D appears to have long-term effects. HDFN was found less severe in subsequent pregnancies of women who had failures of postnatal prophylaxis compared to infants of multiparae women who had no pro-phylaxis^77^. Antenatal prophylaxis given only during first pregnancies, together with postnatal prophylaxis, resulted in a 12-fold reduction in cases with D-immunisation in the second pregnancies^78^. These findings were recently confirmed^79^. It was suggested that the D-immune responses could have been modified by giving anti-D after the responses had started but before they had matured^77,78^. (b) Women with large fetal bleeds (FMH over 20 ml) who were given appropriate doses of anti-D Ig but had persistence of some circulating fetal RBC 6 days after delivery were subsequently found to be protected from D-immunisation, indicating that the immune response had been prevented by the sequestered RBC^80^. (c) IgG anti-Kell (K) injected into K- D- subjects after immunisation with K+ D+ RBC gave a 10-fold reduction in anti-D responders, compared to a control group not given anti-K. This demonstrated that after rapid clearance of RBC to the spleen, antibody-mediated immune suppression is not antigen specific but cell-specific, inhibiting antibody formation to all antigens on the RBC^81^. (d) Besides destruction of the RBC by anti-D Ig, another potential mechanism may be suppression of primed antigen-specific B cells by co-cross-linking B cell receptors (binding RBC antigens) and inhibitory FcγRIIb (with anti-D Ig) (reviewed in^76^). Of note, it was reported that the YB2/0 form of a mAb-D, T125, had greater interactions with both FcγRIIIa and FcγRIIb than the CHO form, thus indicating that low fucosylated anti-D would be effective in this mechanism of B cell suppression^82^ as well as in rapid RBC clearance^29^. (e) Other “non-specific” immunomodulatory effects of prophylactic anti-D could be caused by the anti-D or many other alloantibodies in the donor pool of immuno-globulins (similar to IVIG) using these mechanisms, such as reductions of anti-Fy^a^ in a case report^83^ and of anti-HLA sensitisation in a large survey^84^. HLA class I antigens (Bg) are expressed on most cells including RBC of some normal donors^85^. (f) Animal models, unfortunately, are generally unsatisfactory for understanding anti-D prophylaxis; experiments in immunocompetent mice using xenogeneic cells or glycoproteins elicit innate and/or inflammatory reactions, quite unlike allogeneic RBC and anti-D Ig in humans^76^.

Inflammatory responses must be avoided for RhD prophylaxis. If inflammation accompanies RBC destruction, splenic macrophages mature to DCs, present antigen to T helper (Th) cells and initiate antibody responses to allogeneic proteins^18^. Understandably, it must not occur with mAb-Ds or immune anti-D may be produced.

Several factors may cause inflammation. (a) Pregnant women have strong systemic immunity with mild inflammation^10,11^ and skewing towards antibody (Th2) responses^86^ whilst maintaining local (uterine) tolerance to the fetus^13^. They make robust alloantibody responses to small volumes of allogeneic blood. Consequently, most protein blood groups on RBC and alloantigens on platelets were discovered by investigating cases of HDFN and fetal and neonatal alloimmune thrombocytopenia (FNAIT). (b) Recognition of cells by innate immune receptors may induce phagocytosis accompanied by inflammatory cytokines, promoting antigen presentation; this was observed experimentally for RBC immunisation^87^. (c) Extracellular haemolysis liberates haemoglobin, its breakdown products induce systemic inflammatory responses (febrile reactions and cytokine storms) which can be dangerous. Haemolysis underlies the pathology of HDFN, delayed haemolytic transfusion reactions, and rare reactions of patients with idiopathic thrombocytopenia treated with anti-D^88^, all occurring when the phagocytic capacity of splenic macrophages is saturated and RBC are haemolyzed extracellularly.

Anti-D prophylaxis may be mediated or influenced by cytokines but data are limited. Interleukin (IL)-1Ra, an anti-inflammatory cytokine, was detected during monocyte phagocytosis of BRAD3lab-b-opsonised RBC *in vitro*^89^. Modest increases of tumour necrosis factor-α but not interferon-γ (both pro-inflammatory) were observed briefly (at 4 h) after infusion of RBC coated with Rhophylac 300 or R297-yb2/0^29^. After antenatal pro-phylaxis, slight reductions of IL-1Ra (pro-inflammatory effect) were observed in plasma of 7 of 10 women while modest increases of transforming growth factor-β1 and prostaglandin E2 (immunoregulatory) were recorded in 7 and 5 of these women, respectively^90^. However, no tests to detect fetal cells (FMH) were performed so it is possible these changes in 3 of the 17 cytokines tested^90^ were due to the immunoglobulin component, known to have immunomodulatory effects.

Great care must be taken to ensure the safety and efficacy of mAb-Ds at preventing D-immunisation before trials are performed in pregnant (and postpartum) women. First, anti-D responses are slow and low titre; half of the women immunised during pregnancy produce serologically detectable anti-D by six months post-partum and half of them in subsequent pregnancies, presumably after FMH^91^. Gunson *et al*. proposed this involves slow protracted phagocytosis of fetal RBC as they become effete^92^. Second, normal adults do not have the enhanced humoral immunity of pregnant women; anti-D developed in 50% of subjects only after 2–5 injections of D-positive RBC and rapid clearance of these RBC often occurred before anti-D was detected serologically^93^. Therefore, in the early clinical trials of anti-D, subjects were re-immunised with D-positive RBC several times between 6 and 12 months^33–35,94^. Primary and secondary anti-D responses were detectable 2–4 months or 1–4 weeks after re-immunisation, respectively. This determines which subjects were (a) D-immunised by the first injection of RBC (failure of prophylaxis), (b) D-immunised after RBC challenge (protection by prophylaxis) and (c) non-responders who never make anti-D (non-informative).

Thus both appropriate clinical testing and anti-D glycosylation are required for success with prophylactic mAb-Ds. The previously published clinical trial data of the anti-Ds in this study can be summarised. *Anti-D Ig*: high ADCC, very rapid RBC clearance, prevented D-immunisation. *B mAb-Ds:* (BRAD3, BRAD5) medium ADCC, fast clearance, prevented D-immunisation in 93% subjects, insufficient dose. *HH mAb-Ds:* (Fog1, AD1, G7, G12) low ADCC, variable and slow clearance, stimulated D-immunisation. *CHO mAb-Ds*: (BRAD3, BRAD5, MonoRho) low ADCC, slow and variable clearance, MonoRho may have prevented D-immunisation but this is not proven. *YB2/0 mAb-Ds*: (Fog1, Fog1Δnab, R297) very high ADCC, extremely rapid clearance.

Unfortunately, after much work over three decades, none of the mAb-Ds in this study and also Sym001-cho (Rozrolimupab)^95^ are still in clinical development for prophylaxis against HDFN although the results of prevention of D-immunisation in a phase II/III trial of Roledumab-yb2/0 (R297 with low fucosyl transferase)^96,97^ are awaited with great interest.

Prophylaxis against FNAIT has been proposed and anti-HPA-1a immunoglobulin is being prepared from women immunised by pregnancy for trials^98^. Because anti-HPA-1a is rarely produced after platelet transfusion^99,100^, donors could not be immunised for anti-HPA-1a immunoglobulin, thus monoclonal anti-HPA-1a would be needed for prophylaxis^101^.

This study has shown that the biological activity of mAb-Ds is defined by their producer cell lines. Our results indicate that glycosylation is likely a key determinant of clinical effectiveness. The optimal glycosylation for prophylactic mAb-Ds (and monoclonal anti-HPA-1a) might be different from that of highly cytotoxic monoclonal antibodies for cancer therapy^102^, because haemolysis and inflammation must be avoided. Therefore, to copy and replace anti-D Ig and to prevent possible adverse effects, galactosylation should be over 60% with G2 > G1 > G0 to enhance functional activity. However, lower fucosylation (under 60%) than in most prophylactic anti-Ds^25^ would promote efficient FcγRIIIa interactions and rapid RBC clearance at low mAb-D concentrations. From the available data, these limits may be the best current estimate for reliable, potent mAb-Ds. Of note, abundant supplies of safe, effective and affordable mAb-D are urgently needed to reduce the global burden of HDFN.

## Materials and Methods

### IgG antibodies

mAb-Ds were produced from human EBV-transformed B-lymphoblastoid cell lines (B), mouse myeloma cell lines (NS0 or mouse/human heterohybridomas (HH) formed by fusion of B and P3X63Ag8.653 cell lines), Chinese hamster ovary (CHO) cell lines and rat myeloma YB2/0 or rat/human heterohybridoma cell lines. All mAb-Ds were IgG1 except BRAD3 (IgG3). The following individuals submitted antibodies for glycosylation analysis: Sylvia Miescher, (Rhophylac 300 (300 µg, 1500 IU, 2 ml) anti-D Ig (Quality Control Grade, Lot 02905-00092) and MonoRho-cho); Rosey Mushens, (BRAD3lab-b, BRAD5lab-b, JAC10-b, AB5-b, Fog1-hh); Joan Dalton, (BRAD3clin-b, mBRAD3-b, rBRAD3-cho, BRAD5clin-b, mBRAD5-b, rBRAD5-cho); Natalia Olovnikova (G7-b, G7-hh, G12-b, G12-hh, G12-yb2/0 (rat/human), G108-b); Christof de Romeuf, (AD1-hh, R297-yb2/0); Kathryn Armour (Fog1-ns0, Fog1-yb2/0 and Fog1Δnab-yb2/0^32^). BRAD3lab-b and BRAD5lab-b were prepared from low cell density flask cultures for experimental use and BRAD3clin-b and BRAD5clin-b were produced for clinical testing^33^ in high cell density hollow fibre bioreactors; mBRAD3-b, mBRAD5-b, rBRAD3-cho and rBRAD5-cho were subsequently also produced from hollow fibre bioreactors^31^. IVIG (Hepatect CP 50 IU/ml) was kindly provided by P. Griffiths from Biotest (UK) Ltd, Birmingham, UK.

### Purification and quantification of IgG anti-D

Anti-D was affinity purified from Rhophylac 300 anti-D Ig^44^. IgGs from this RBC eluate and from culture supernatants of mAb-Ds were purified using Protein G and IgG concentrations determined by ELISA. Only IgG1 was detected in anti-D purified from Rhophylac 300 (at 10 μg/mL) by haemagglutination with anti-IgG subclass mAbs^44^. The anti-D fraction termed Rhophylac was used in this study.

### Glycosylation analysis 1: N-glycan analysis in NIBRT GlycoScience Group, Dublin (Flu)

Antibodies were reduced, alkylated and N-glycans were released from IgG heavy chain from SDS-PAGE gel bands by digestion with N-glycosidase F (PNGase F, Prozyme, San Leandro, CA) as described by Royle *et al*.^103^. Briefly, gels were washed and N-glycans were released by PNGase F. Released N-glycans were fluorescently labelled with 2-aminobenzamide (2-AB) by reductive amination using a LudgerTagTM 2-AB labelling kit (Ludger Ltd., Abingdon, UK) and excess of 2-AB was removed by paper chromatography^103^.

Labelled glycans were analysed by 3 hours normal phase high-performance liquid chromatography **(**NP-HPLC) using a TSK-Gel Amide-80 4.6 × 250 mm column (Anachem, Luton, UK) on a 2695 Alliance separations module (Waters, Milford, MA) equipped with a Waters temperature control module and a Waters 2475 fluorescence detector. Solvent A was 50 mM formic acid adjusted to pH 4.4 with ammonia solution. Solvent B was acetonitrile. Gradient conditions were a linear gradient of 20–58% A, over 152 min at a flow rate of 0.4 mL/min. Samples were injected in 80% acetonitrile^103^. Fluorescence was measured at 420 nm with excitation at 330 nm. The system was calibrated using an external standard of hydrolysed and 2AB-labeled glucose oligomers to create a dextran ladder, as described previously^103^. NP-HPLC chromatograms generated from the samples are in Supplementary Fig. S1.

For exoglycosidase digestion of 2-AB labelled N-glycans, enzymes were supplied by Prozyme. The 2AB-labelled glycans were digested in a volume of 10 μL for 18 h at 37 °C in 50 mM sodium acetate buffer, pH 5.5 (except in the case of jack bean α-mannosidase (JBM) where the buffer was 100 mM sodium acetate, 2 mM Zn^2+^, pH 5.0), using arrays of the following enzymes: *Arthrobacter ureafaciens* sialidase (ABS, EC 3.2.1.18), 0.5 U/mL; *Streptococcus pneumoniae* sialidase (NAN1, EC 3.2.1.18), 1 U/mL; coffee bean alpha galactosidase (CBG, EC 3.2.1.22), 25 U/mL; bovine testes β-galactosidase (BTG, EC 3.2.1.23), 1 U/mL; bovine kidney alpha-fucosidase (BKF, EC 3.2.1.51), 1 U/mL and JBM (EC 3.2.1.24), 60 U/mL. After incubation, enzymes were removed by filtration through 10 kDa protein-binding EZ filters (Millipore Corporation)^103^. N-glycans were assigned using exoglycosidase digestions (Supplementary Table S2) and Glycobase and features outlined in Tables 1–3 were calculated based on these assignments (Supplementary Table S2).

### Glycosylation analysis 2: N-glycan analysis at Leiden University Medical Center

#### IgG Total N-glycosylation analysis (Eth)

After protein denaturation, N-glycans were released with 1 mU recombinant peptide-N-glycosidase F (PNGase F; Roche Diagnostics, Mannheim, Germany) at 37 °C overnight as described previously^104,105^. The selective ethyl-esterification of 2,6-linked sialic acids and lactonization of 2,3-linked sialic acid was performed on the released N-glycans^106^, followed by glycan purification by HILIC-SPE using cotton as stationary phase^107^ and glycan elution with 10 µl of water. For MALDI-TOF-MS analysis, samples were spotted on an AnchorChip MALDI target (Bruker Daltonics, Bremen, Germany) together with sodiated (1 mM NaOH) Super-DHB (Sigma-Aldrich) matrix. All analyses were performed on an UltraFlextreme MALDI-TOF/TOF-MS equipped with a Smartbeam II laser (FlexControl 3.4 Build 119, Bruker Daltonics). The MS was operated in reflectron positive (RP) ion mode, calibrated on the known masses of a peptide calibration standard (Bruker Daltonics). For sample measurements 10000 laser shots were accumulated at a laser frequency of 1000 Hz, using a complete sample random walk with 200 shots per raster spot. Tandem mass spectrometry (MALDI-TOF/TOF-MS/MS) was performed on mostly sialylated variants of IgG glycans via laser-induced dissociation, and compositions as well as structural features of N-glycans were confirmed on the basis of the observed fragment ions (not shown).

Spectra were exported as text and subjected to recalibration and data extraction using an in-house developed Python script. Glycan peaks were detected and extracted using a signal/noise cut-off of 3. Total glycan intensity per spectrum was normalised to 100%, and derived traits were calculated based on the compositional features (Supplementary Tables S3 and S4) (hexose = H; N-acetylhexosamine = N; fucose = F; α2,6-linked N-acetylneuraminic acid = E; α2,3-linked N-acetylneuraminic acid = L; α2,6-linked N-glycolylneuraminic acid = Ge; α2,3-linked N-glycolylneuraminic acid = Gl).

### Glycosylation analysis 3: IgG Fc glycopeptide analysis (N-glycosylation) at Leiden University Medical Center (GP)

IgG was enzymatically digested with trypsin and analysed by reverse phase-nanoLC-MS. Electrospray ionisation was achieved with a CaptiveSpray nanoBooster (Bruker Daltonics) using acetonitrile-enriched nitrogen gas to enhance sensitivity. Glycopeptides were detected using a quadrupole-time-of-flight (TOF) mass spectrometer (MS) (maXis impact HD ultra-high resolution QTOF; Bruker Daltonics)^108^. Double and triple charged tryptic Fc glycopeptide signals were integrated and normalised to the subclass-specific total glycopeptide intensity. Quality of mass spectra was evaluated based on intensities of total IgG1 glycoforms. Glycosylation traits were calculated as detailed in Supplementary Table S3.

### Analysis of mAb-D glycosylation in small scale earlier studies

Methods used for other studies reported in Supplementary Table S1 were MALDI-TOF-MS analysis of IgG1 Fc-glycopeptides^25^, analysis by HVE-AEC, gel filtration chromatography and Concanavalin A binding of oligosaccharides released by hydrazinolysis^109^, quantitation of % G0 by binding of GlcNAc-specific mAb GN7^59^, chromatographic separation of fluorescently labelled neutral oligosaccharides^49^, enzymatically released glycans analysed by HPCE-LIF^82^ and FAB-MS and MALDI-MS of permethylated N-glycans (Carbohydrate structure of rBRAD-3 and rBRAD-5; Joan Dalton, BioProducts Laboratory, UK, email, September 26, 2007; permission to publish subsequently given).

### ADCC assay

Peripheral blood mononuclear cells (PBMC) depleted of adherent monocytes were incubated in triplicate for 16 h at 37 °C with papainized ^51^Cr-labelled group OR_1_R_2_ RBC (15:1 ratio) and anti-D in RPMI1640 containing 3% AB serum (to block FcγRI on residual monocytes)^50^ and 7% fetal calf serum; after centrifugation, radioactivity was determined in aliquots of supernatant^72^. The percent specific lysis (% haemo-lysis) was calculated as: % specific lysis = 100 × (experimental release – spontaneous release)/(maximum release – spontaneous release). To confirm FcγR utilisation, lysis by IgG1 anti-D was blocked by anti-FcγRIII (3G8) but not by anti-FcγRII (IV.3)^50^.

### Analysis of data from previous clinical trials of RBC clearance and prevention of D-immunization

The efficacy of prophylactic anti-D Ig depends on removal of fetal RBC from the circulation by 72 hours^110^. Clinically, tests for FMH are performed to determine whether fetal D-positive RBC have been cleared by this time^111^. For initial clinical trials, pre-menopausal women are not enrolled because they might become D-immunised which could lead to HDFN in subsequent pregnancies. Early studies showed that if anti-D prevented D-immunisation in men, it would be suitable for prophylaxis in women. Using healthy male volunteers, eight mAb-Ds had been tested in four autologous RBC clearance studies and seven mAb-Ds in five allogeneic RBC clearance studies. All the trial protocols varied; details of methods, ethics approval and informed consent are given in the original papers cited in Tables 4 and 5. Autologous RBC clearance measured the extent of radioactivity remaining in blood of D-positive subjects after injection of their *ex vivo* ^51^Cr labelled RBC coated with anti-D. Study periods were between 1 h and 6 days after injection. Allogeneic studies measured clearance of D-positive RBC (labelled with ^51^Cr or detected by flow cytometry) injected into D-negative recipients before (simulating postnatal prophylaxis) or after (equivalent to antenatal prophylaxis) anti-D administration, with blood samples taken up to 7 days. To assess whether mAb-D could prevent D-immunisation, these subjects were then tested regularly (every 2 or 4 weeks) for 6 months to detect anti-D responses (indicating failure of prophylaxis). In studies of B mAb-Ds, subjects were then re-immunised with D-positive RBC at 6 and 9 months and tested regularly up to a year to determine which were responders to these unprotected immunisations who had been prevented from making anti-D after the first RBC injection by mAb-Ds.

## Supplementary information

Supplementary Information.

Supplementary Table S2.

Supplementary Tables S3 and S4.

## Data Availability

All data generated or analysed during this study are included in this published article and its Supplementary Information files. Not all mAb-Ds (antibodies or cell lines) may be available due to being produced many years ago in laboratories that have since ceased working on them or closed down.
