# Supplementary material for: Anti-D monoclonal antibodies from 23 human and rodent cell lines display diverse IgG Fc-glycosylation profiles that determine their clinical efficacy
Source: Sci Rep. 2020 Jan 30;10:1464. doi: 10.1038/s41598-019-57393-9 (PMC6992666; doi:10.1038/s41598-019-57393-9)
Supplement: Supplementary file 1 — Supplementary Information. [file 41598_2019_57393_MOESM1_ESM.pdf]

Anti-D monoclonal antibodies from 23 human and rodent cell lines display diverse IgG Fc-glycosylation profiles that determine their clinical efficacy

Belinda M. Kumpel, Radka Saldova, Carolien A.M. Koeleman, Jodie L. Abrahams, Agnes Hipgrave Ederveen, Kathryn L. Armour, Natalia I. Olovnikova, Gestur Vidarsson, Rick Kapur, Pauline M. Rudd & Manfred Wuhrer

Supplementary Table S1

Comparison of glycosylation data (in bold) with findings of earlier studies (in italics)

Reference	Anti-D	% Sialyl-ation	% Galacto-sylation	% G0	% G1	% G2	% Fucosyl-ation	% Bisected
	**Rhophylac**		**84**				**80**	**12**
25	*Rhophylac*		*81*				*82*	*6*
	**BRAD3lab-B**	**29**	**69**	**11**	**35**	**51**		
109	*BRAD3lab-B*	*15*	*87*	*2*	*21*	*77*		
59	*BRAD3lab-B*			*2*				
	**BRAD3clin-B**	**32**	**61**	**16**	**39**	**41**		**62**
109	*BRAD3clin-B*	*26*	*59*	*11*	*59*	*30*		*51*
	**mBRAD3-B**	**28**					**79**	**64**
Dalton	*mBRAD3-B*	*20*					*high*	*80*
	**BRAD5lab-B**			**2**				
59	*BRAD5lab-B*			*4*				
	**BRAD5clin-B**	**34**	**64**	**12**	**42**	**43**		**66**
109	*BRAD5clin-B*	*21*	*64*	*8*	*56*	*36*		*34*
	**mBRAD5-B**	**24**					**90**	**42**
Dalton	*mBRAD5-B*	*20*					*high*	*40*
	**JAC10-B**	**46**	**84**	**2**	**24**	**72**		
109	*JAC10-B*	*21*	*83*	*5*	*24*	*71*		
	**G12-B**	**37**	**84**	**1**	**30**	**69**	**95**	
49	*G12-B*	*33*	*56*				*high*	
49	*G108-B*	*26*					*high*	
49	*Anti-D Ig*	*30*	*high*	*7*	*23*	*41*	*medium*	
	**rBRAD3-CHO**	**5**	**33**	**49**	**33**	**16**	**74**	**0**
Dalton	*rBRAD3-CHO*	*5*	*36*	*45*	*38*	*17*	*high*	*0*
	**rBRAD5-CHO**	**4**	**26**	**56**	**37**	**7**	**91**	**0**
Dalton	*rBRAD5-CHO*	*5*	*31*	*49*	*41*	*10*	*high*	*0*
82	*T125(R297)-CHO*		*26*	*56*	*37*	*7*	*81*	
	**R297-YB2/0**		**45**	**29**	**52**	**19**	**34**	
82	*T125(R297)-YB2/0*		*41*	*33*	*53*	*14*	*32*	

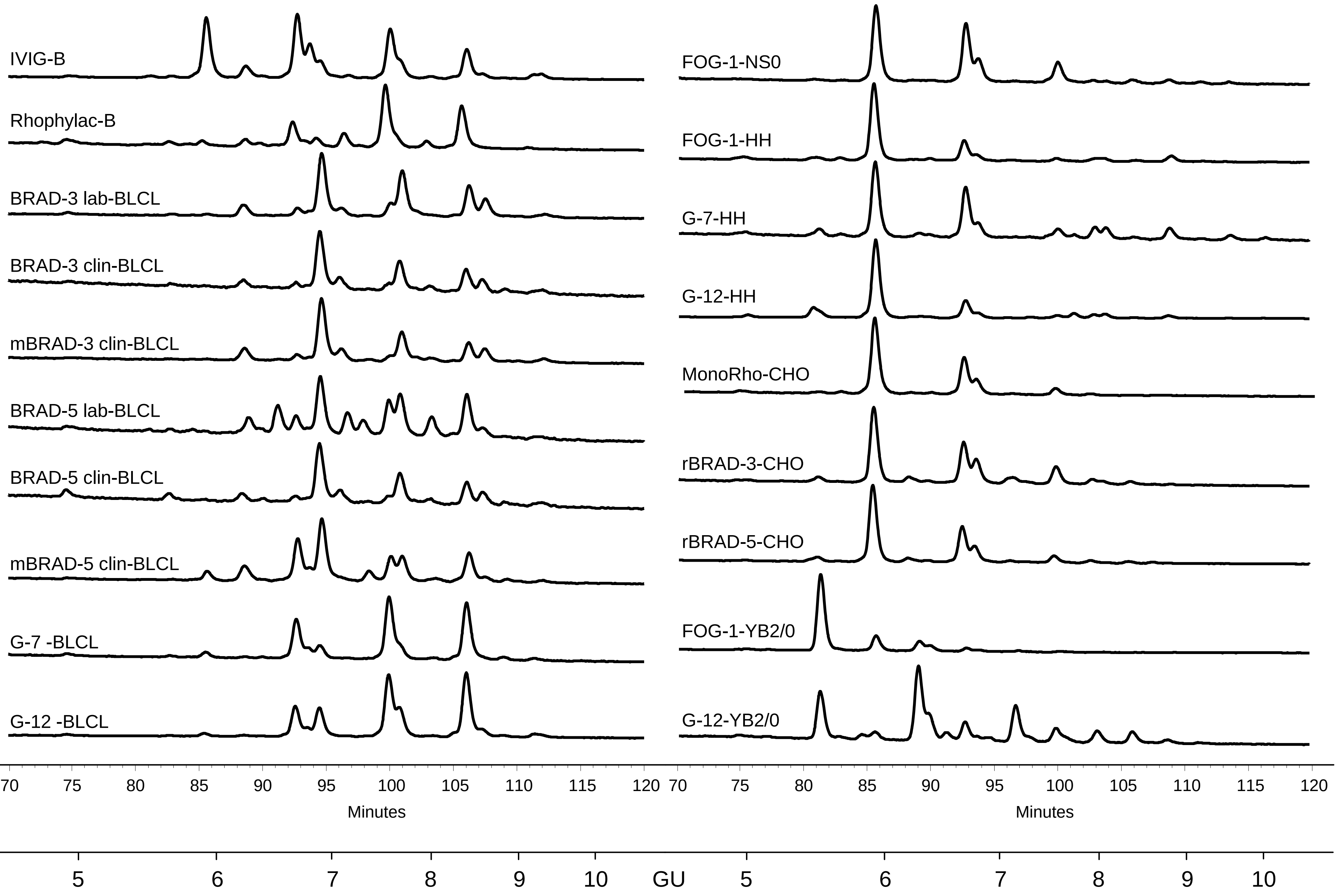

Figure S1. NP-HPLC chromatograms of released N-glycans from IVIG and anti-Ds
